# 

**Published:** 1890-05-10

**Authors:** 


### NEW OFFICES IN THE STRAND.

The Hospital *is now published at* 140, Strand, W.C. *It has been found desirable to establish* The Hospital *in this central and convenient locality, owing to the rapid extension of the business of this paper of late.*

### NOTICE TO CORRESPONDENTS.

All MS., Letters, Books for Review, and other matters intended for the Editor should be addressed The Editor, The Lodge, Porchester Square, London, W.

The Editor cannot undertake to return rejected M.S., even when accompanied by stamped directed envelope.

### Notice to Advertisers and Subscribers.

All Advertisements, Orders for Copies of The Hospital, and Business Communications should be addressed to The Publisher, *not to the Editor*, The Hospital, 140, Strand, W.C.

### Bound Volumes of "The Hospital."

Vol. VI., containing full page portraits of T.R.H. the Prince and Princess of Wales, and Miss Florence Nightingale, is obtainable at 4s., post free.

Orders will be received for Vol. VII., 4s. post free. Cases for binding may be had of the Publisher, at 1s. 6d. each.

### To Residents, Secretaries, and Matrons.

The Editor will be glad to have the Regulations and each Report as issued by Asylums, Hospitals, Convalescent and Nursing Institutions, as well as those of other Charities, and an early copy in duplicate of all Appeals and Festival Notices, etc., addressed to *The Editor, The Lodge, Porchester Square, London, W.* Any superintendent, secretary, matron, or other chief official who regularly sends such information may be registered, on application to the Publisher, to receive free of cost a cover for binding The Hospital in half-yearly volumes.

### Scraps and Gleanings.

Lady Rosebery recently inspected Epsom Cottage Hospital.

Some successful tableaux have been given at Bath in aid of the united Hospitals.

Leprosy is abating in New Caledonia. Six lazerettos have been established.

There is reported to be a man in Limerick Workhouse who can fast for 40 days.

The Bazar de la Charité held in Paris last week was a huge affair in which over 70 charities participated.

At a meeting held last week it was decided to hold medical classes at Queen Margaret's College for Women, Glasgow.

Wimbledon Cottage Hospital treated 133 patients last year. There was a very poor attendance at the annual meeting.

Mrs. J. H. Allen is At Home to-day (May 10th), when a discussion will be held as to the best means of helping the Home for Sick Children at Coldash.

The Bazaar held last month at Derby, in aid of the Children's Hospital, yielded a net sum of £1,024. The managers of the bazaar are to be congratulated.

Mr. E. J. Lehman, a millionaire merchant of Chicago, one of the members of the World's Fair committee, has been sent to the Bloomingdale Asylum for the Insane.

A dramatic entertainment will be given at St. George's Hall on May 20th in aid of the Alexander Hospital for Hip Diseases. Tickets can be had from the Lady Superintendent.

In the North Gallery of the Edinburgh Exhibition is an interesting case containing models, photographs, literature, &c., illustrating the world-wide work of the Missions to Seamen Society, 11, Buckingham Street, Strand, London.

### The Students' Column.

### UNIVERSITY OF DURHAM.

#### SECOND EXAMINATION FOR THE DEGREE OF BACHELOR IN MEDICINE.

The following candidates have satisfied the examiners :—

*Honours—First Class.*—Clifford Winslow Turner, M.R.C.S., Yorkshire College, Leeds.

*Honours—Second Class.*—James Alfred Hutton, M.R.C.S., L.R.C.P., Middlesex Hospital.

*Pass List.*—George Henry Vane Appleby, College of Medicine, Newcastle-upon-Tyne; Arthur Badock, London Hospital; William Henry Bishop, College of Medicine, Newcastle-upon-Tyne; Frederick Carden Brodie, M.R.C.S., L.R.C.P., L.S.A., Middlesex Hospital; Thomas Leigh Bryan, College of Medicine, Newcastle-upon-Tyne; Hugh Somerville Burniston, St. Mary's Hospital; Allan Gordon Russell Cameron, St. Mary's Hospital; Henry Augustus Collinson, College of Medicine, Newcastle-upon-Tyne; Arthur James Dale, College of Medicine, Newcastle-upon-Tyne; Taylor Dixon, College of Medicine, Newcastle-upon-Tyne; Charles Chester Eardley-Wilmot, St. Bartholomew's Hospital; John William Henry Eyre, Guy's Hospital; Arthur Allen Fennings, St. Mary's Hospital; William Fowler, College of Medicine, Newcastle-upon-Tyne; Jabez Percival Iredale, College of Medicine, Newcastle-upon-Tyne; Walter Denton Johns, B.A., College of Medicine, Newcastle-upon-Tyne; Hugh Meyrick Jones, College of Medicine, Newcastle-upon-Tyne; William Martin, B.A., College of Medicine, Newcastle-upon-Tyne; Ronald Seymour McPherson, College of Medicine, Newcastle-upon-Tyne; Alworth Edward Merewether, London Hospital; Christopher Michael O'Brien, Trinity College, Dublin; Willie Oliver, College of Medicine, Newcastle-upon-Tyne; James Peacock, College of Medicine, Newcastle-upon-Tyne; Arthur William Port, St. Bartholomew's Hospital; Lionel Litchfield Preston, M.R.C.S., Middlesex Hospital; Edmund Race, College of Medicine, Newcastle-upon-Tyne; John Melville Ritchie, Queen's College, Birmingham; Frederick Robson, College of Medicine, Newcastle-upon-Tyne; Lancelot William Rolleston, St. Bartholomew's Hospital; Noel Florentine Rowstron, St. Bartholomew's Hospital; Harry Shore, Queen's College, Birmingham; Charles Stewart, College of Medicine, Newcastle-upon-Tyne; Ernest Frank Syrett, St. Bartholomew's Hospital; Leslie Cavendish Thorne Thorne, St. Bartholomew's Hospital; William James Nathaniel Vincent, London Hospital; James Powell Willis, College of Medicine, Newcastle-upon Tyne; George Stephen Ware, M.R.C.S., L.R.C.P., L.S.A., Middlesex Hospital.

#### EXAMINATION FOR THE LICENCE IN SANITARY SCIENCE.

The following Candidates have satisfied the Examiners :—John William Hembrough, M.D.; James Hindhaugh, M.B., B.S., Durham; James Archibald Jackson, M.B., Glasgow; James Kelland, M.B., C.M., L.R.C.P., L.R.C.S., Ed.; James Aspinall Marsden, M.R.C.S., Eng.; Nathan Raw, M.B., B.S., Durham.

